# Uncovering homeologous relationships between tetraploid *Agropyron cristatum* and bread wheat genomes using COS markers

**DOI:** 10.1007/s00122-019-03394-1

**Published:** 2019-07-16

**Authors:** Mahmoud Said, Alejandro Copete Parada, Eszter Gaál, István Molnár, Adoración Cabrera, Jaroslav Doležel, Jan Vrána

**Affiliations:** 1 Institute of Experimental Botany, Center of the Region Haná for Biotechnological and Agricultural Research, Šlechtitelů 31, 78371 Olomouc, Czech Republic; 2 Field Crops Research Institute, Agricultural Research Centre, 9 Gamma Street, Giza, Cairo, 12619 Egypt; 3 Genetics Department, ETSIAM, Agrifood Campus of International Excellence (ceiA3), University of Córdoba, 14071 Córdoba, Spain; 4 Agricultural Institute, Centre for Agricultural Research, Hungarian Academy of Sciences, Martonvásár, 2462 Hungary

## Abstract

**Key message:**

Using COS markers, the study reveals homeologous relationships between tetraploid *Agropyron cristatum* and bread wheat to support alien introgression breeding of wheat.

**Abstract:**

Crested wheatgrass (*Agropyron cristatum* L. Gaertn.) is a wild relative of wheat that possesses many genes that are potentially useful in wheat improvement. The species comprises a complex of diploid, tetraploid and hexaploid forms. In this study, wheat–*A. cristatum* chromosome, telosome and translocation lines were used to characterize syntenic relationships between tetraploid *A. cristatum* and bread wheat. Prior to mapping COS markers, the cytogenetic stock lines were characterized for fertility and by FISH and GISH for karyotype stability. Out of 328 COS markers selected for the study, 279 consistently amplified products in tetraploid *A. cristatum*, and, out of these, 139 were polymorphic between tetraploid crested wheatgrass and wheat. Sixty-nine markers were found to be suitable for the detection of tetraploid *A. cristatum* chromosomes 1P–6P in wheat, ranging from 6 to 17 markers per chromosome. BLASTn of the source ESTs resulted in significant hits for 67 markers on the wheat pseudomolecules. Generally, COS markers of the same homeologous group were detected on similar arms in both *Agropyron* and wheat. However, some intragenomic duplications and chromosome rearrangements were detected in tetraploid *A. cristatum*. These results provide new insights into the structure and evolution of the tetraploid *A. cristatum* genome and will facilitate the exploitation of the wild species for introgression breeding of bread wheat.

**Electronic supplementary material:**

The online version of this article (10.1007/s00122-019-03394-1) contains supplementary material, which is available to authorized users.

## Introduction

The gene pool of bread wheat (*Triticum aestivum* L., 2*n* = 6*x* = 42, AABBDD) was narrowed down during thousands of years of domestication, cultivation and breeding. The dwindled genetic diversity hampers the development of cultivars with improved quality and tolerance to biotic and abiotic stresses. As wild crop relatives were not subjected to human selection, they exhibit large genetic variation and represent an attractive source of alleles and genes for crop improvement (Tanksley and McCouch 1997).

The genus *Agropyron* includes 10–15 species (Asay and Jensen 1996; Martín et al. 1999; Liu et al. 2010) and is a remarkable source of new gene variants for wheat improvement (Asay and Johnson 1990; Limin and Fowler 1990; Dong et al. 1992; Friebe et al. 1992). The most widespread species of the genus is *A. cristatum* (L.) Gaertn (Yang et al. 2014), also known as crested wheatgrass, which is a perennial, facultatively allogamic and autocompatible species. *Agropyron cristatum* contains the genome P in a series of diploid (2*n* = 2*x* = 14), tetraploid (2*n* = 4*x* = 28) and hexaploid (2*n* = 6*x* = 42) forms (Löve 1982, 1984; Dewey 1984; Li et al. 2007). The diploids are less common and distributed from Europe to Mongolia, whereas tetraploids are widespread, in central Europe, the Middle East and central Asia. Hexaploids are rare and are mainly found in Turkey, Iran and Georgia (Dewey and Asay 1982; Copete et al. 2018). The nature of the polyploid genome of *A. cristatum* and the origin of its P genome(s) have not yet been clarified. Schulz-Schaeffer et al. (1963) proposed the segmental allopolyploid nature of tetraploid and hexaploid *A. cristatum*, while other authors consider the tetraploid *A. cristatum* as autopolyploid, originating from the diploid *A. cristatum* (Taylor and McCoy 1973; Vogel et al. 1999; Zhao et al. 2017).

A number of agronomically important traits were identified in *Agropyron* spp., including resistance to pests and diseases such as barley yellow dwarf virus (Sharma et al. 1984; Shukle et al. 1987), wheat streak mosaic virus (Sharma et al. 1984; Brettell et al. 1988; Triebe et al. 1991), yellow rust, leaf rust and stem rust (Knott 1964, 1968; Cauderon and Rhind 1976; Whelan 1988; Friebe et al. 1992; Zhang et al. 2017), powdery mildew (Copete and Cabrera 2017) and tolerance to different abiotic stresses like cold (Limin and Fowler 1987), salinity (Dewey 1960, 1962; McGuire and Dvořák 1981; Forster et al. 1987; Littlejohn 1988) and drought (Dewey 1984) and genes affecting yield (Song et al. 2013). These traits would be desirable to transfer into wheat by interspecific hybridization. Interspecific hybridization is a promising tool to utilize the extant genetic diversity in wheat improvement through the chromosome-mediated transfer of useful agronomic traits (Feuillet et al. 2008). However, the crossability of diploid *A. cristatum* with wheat is low due to the disequilibrium in the endosperm balance number in the hybrid seeds (Chen et al. 1989), while hybridization between wheat and tetraploid *A. cristatum* can be easily done (Chen et al. 1989; Martín et al. 1999). As the crosses between wheat and hexaploid *A. cristatum* have not been reported yet, the tetraploid form is the most widely used crossing partner in the wheat-crested wheatgrass introgression breeding programs (Chen et al. 1989, 1994; Li et al. 1997, 1998, 2016; Soliman et al. 2007; Han et al. 2014; Ochoa et al. 2015).

In order to make *A. cristatum* genes accessible for wheat breeding programs, a set of wheat–*A. cristatum* (1P–6P) disomic addition lines has been developed by Chen et al. (1989, 1994) and Han et al. (2014) together with the production of ditelosomic additions 2PS, 2PL, 4PS, 5PL, 6PS or 6PL (Chen et al. 1989, 1994). Moreover, production of wheat–*A. cristatum* translocation lines was reported by Luan et al. (2010) and Song et al. (2013). In these studies, the chromosome 6P has been identified as the carrier of genes controlling yield components including the number of florets and kernels per spike (Luan et al. 2010) and of a locus conferring resistance to stripe rust (Zhang et al. 2017). Ochoa et al. (2015) developed another line, TH4, which carries a Robertsonian translocation involving the long arm of wheat chromosome 1B and the short arm of an unidentified tetraploid *A. cristatum* chromosome, with substantial resistance to leaf rust. The effectiveness of the gene transfer can be facilitated by high-throughput tools suitable for screening backcross populations for the presence of alien introgression lines with desired karyotype. On the other hand, the main selection tools are laborious cytogenetic methods, such as C-banding (Friebe et al. 1996), fluorescence in situ hybridization (FISH) (Rayburn and Gill 1985; Schwarzacher and Heslop-Harrison 2000; Schneider et al. 2005) and genomic in situ hybridization (GISH) (Schwarzacher et al. 1989; Le et al. 1989). The potential of FISH to identify particular chromosomes and their segments is also compromised by the lack of suitable probes, and the cytogenetic methods suffer from low sensitivity to detect small introgressed segments (Choi et al. 2009).

The efficiency of the gene transfer from wild relatives to wheat also depends on the homology between wheat and alien chromosomes. If collinearity between the donor and recipient genomes is broken down due to evolutionary chromosome rearrangements, homeologous recombination may result in progenies with nonbalanced genomes (Devos et al. 1993; Zhang et al. 1998). Altered structure of the donor chromosomes may interfere with meiotic recombination and hamper attempts to reduce the size of introgressed chromatin to eliminate undesirable traits (Nasuda et al. 1998). A further consequence of chromosome rearrangements, the genes on alien chromosome segments do not compensate for the loss of wheat genes and thus may negatively affect the agricultural performance of wheat–alien translocations. Polyploidization may induce genome rearrangements (Ma et al. 2004; Han et al. 2005, 2017; Zhang et al. 2013) and in case of *A. cristatum* Han et al. (2014) found that chromosome 6P of the tetraploid form differs from its wheat homeologous by large rearrangements. Later, Said et al. (2018) used single-gene FISH to investigate wheat–*A. cristatum* homeologous relationships and found several chromosomal rearrangements in diploid *A. cristatum* relative to wheat. However, the knowledge on the P genome structure of tetraploid *A. cristatum* remains poor and the mentioned observations underline the need for a structural comparison of P chromosomes in tetraploid *A. cristatum* with those of wheat.

Molecular markers have higher throughput than cytogenetic methods and capable of detecting small segments of *A. cristatum* chromatin; thus, they would facilitate the alien introgression breeding of wheat (Copete and Cabrera 2017). However, specific expressed sequence tag (EST) markers have been developed only for chromosomes 6P (Cheng et al. 2012) and sequence-tagged site (STS) markers for 7P (Lu et al. 2016) of tetraploid *A. cristatum*. Thus, the low coverage of P genome with small number of molecular markers limits the efficiency of introgression breeding and the investigation of wheat–tetraploid *A. cristatum* homeologous relationships. The chromosome-based genome assembly is still not available in species with large and complex genomes such as *A. cristatum* with a 1C genome size of 6352 Mbp (Said et al. 2018), a fact that strongly limits the development of DNA markers for the P genome. An alternative strategy is exploiting molecular markers from genetically related species and their assignment to individual chromosomes of the donor genome using alien introgression lines (Said and Cabrera 2009; Cherif-Mouaki et al. 2011; Said et al. 2012; Copete and Cabrera 2017).

Conserved orthologous set (COS) genes (> 1000) are well preserved throughout evolution in both sequence and copy number between wheat and the model species rice and *Brachypodium* (Quraishi et al. 2009). COS markers were designed over the exon–intron boundaries of orthologous genes. As introns sequences are less conserved than exons (Yu et al. 2005), COS markers are potentially polymorphic. The polymorphism can be detected as a difference in the size of PCR products amplified from wheat and related species. In agreement with this, COS markers are highly transferable between species such as rice, wheat, maize, sorghum, barley and several species of *Aegilops* (Parida et al. 2006; Burt and Nicholson 2011; Howard et al. 2011; Molnár et al. 2013, 2016). Wheat-specific COS markers are also transferable to diploid wheatgrass species, such as *Thinopyrum bessarabicum* (Savul.&Rayss) A. Löve, *Pseudoroegneria spicata* (Pursh) A. Löve and *A. cristatum* (Linc et al. 2017). Because this marker system is based on orthologous genes, COS markers enable the comparison of orthologous regions on the chromosomes of related species. Using COS markers on wheat–*Aegilops* disomic addition lines, Molnár et al. (2013) assigned a total of 132 and 156 loci to the U and M genome chromosomes of *Aegilops* and investigated the homeologous relationships between the U and M genomes of *Aegilops* and wheat. Later, Gaál et al. (2018) assigned sixty COS markers to the E-genome chromosomes of *Thinopyrum elongatum* revealing several chromosomal rearrangements relative to wheat.

Motivated by the need to improve the efficiency of the chromosome-mediated gene transfer from tetraploid *A. cristatum* into wheat, the aim of the present study was to increase the number of markers suitable to detect P chromosomes in the wheat genetic background and to study orthologous relationships between the chromosomes of *A. cristratum* and bread wheat. Using PCR with DNA from a wheat–*A. cristatum* disomic addition lines 1P, 2P, 3P, 4P, 5P and 6P, and wheat–*A. cristatum* telosome addition lines 2PS, 2PL, 4PS, 5PL, 6PS and 6PL (Chen et al. 1989, 1994), we assigned wheat-specific COS markers to the chromosomes and chromosome arms of *Agropyron*. Sequence similarity search of the source ESTs of the *Agropyron* specific COS markers on the wheat pseudomolecules has also been done to investigate wheat–*Agropyron* genome relationships.

## Materials and methods

### Plant material

**Fig. 1:**
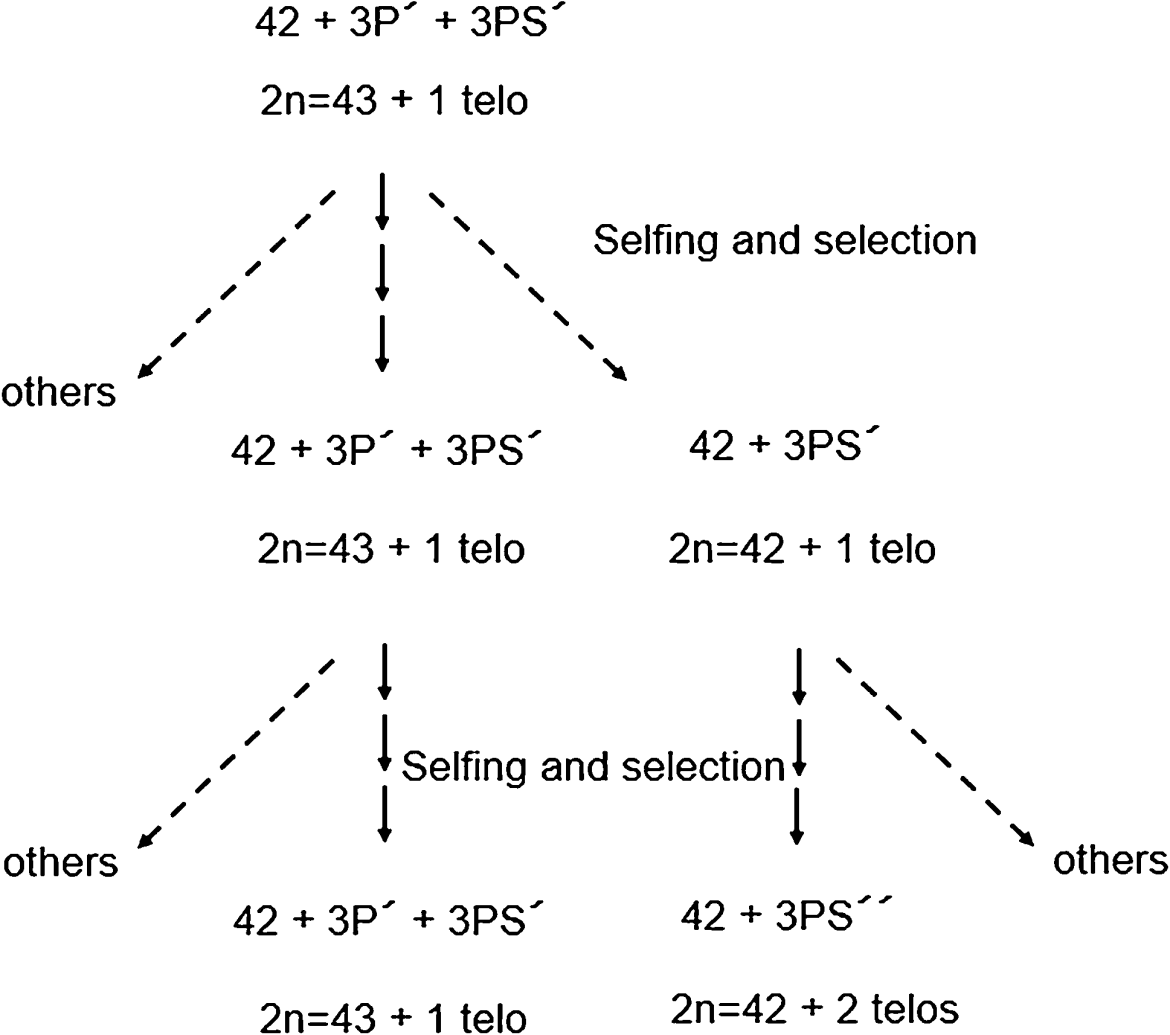
The breeding procedure used in this study to obtain the ditelosomic addition chromosome short arm 3PS from tetraploid *A. cristatum* in the genetic background of wheat CS

The seeds of diploid *A. cristatum* cv. Parkway (2*n* = 2*x* = 14, PP), accession number PI 415799 were provided by Dr Joseph Robins (ARS Forage and Range Research Laboratory, USDA, Logan, USA). The seeds of tetraploid *A. cristatum* (2*n *= 4*x *= 28, PPPP) accession PI 222957 were obtained from the USDA genebank (https://npgsweb.ars-grin.gov/gringlobal/search.aspx). The seeds of parental wheat cv. Chinese Spring (CS) together with the seeds of CS-*A. cristatum* chromosome addition lines (CS-P) carrying chromosomes 1P, 2P, 3P, 4P, 5P and 6P (CS-1P–6P), telosome addition lines (CS-PS, PL) for chromosome arms 2PS, 2PL, 4PS, 5PL, 6PS and 6PL were produced by Chen et al. (1989, 1994) were also used in the present study as well as the CS-*A. cristatum* Robertsonian translocation line (CST-P) involving the long arm of wheat chromosome 1B and the short arm of an unidentified chromosome from tetraploid *A. cristatum* (Ochoa et al. 2015). The new CS-*A. cristatum* telosome addition line 3PS (CS-3PS) was developed in the present study at the Institute of Experimental Botany (Olomouc, Czech Republic) by successive selfing the line CS-3P possessing one 3P chromosome and one P telosome and selecting for the genotypes carrying mono- or ditelosomic 3PS (Fig. 1).

### Karyotype stability, fertility and morphological characteristics

Before the COS marker analysis, all wheat–*A. cristatum* chromosome and telosome addition lines and the translocation line were characterized for fertility and karyotype stability during three successive generations. Fertility and spike morphological characteristics were estimated from five spikes for the following traits: spikelets per spike, seeds per spike and spike length. An analysis of variance was carried out, and a mean values comparison was accomplished using the least significant difference method (*P* ≤ 0.05). Statistical analysis was performed with Minitab 18 (www.minitab.com). Karyotype stability of the wheat–*A. cristatum* lines was evaluated by chromosome counting and genomic in situ hybridization (GISH) as detailed in Szakács and Molnár-Láng (2010). Chromosome compositions of the wheat–*A. cristatum* lines over the generations were expressed in percent (%): the number of plants with a specific chromosome composition divided by the total number of plants analyzed from a specific line and multiplied by 100.

### Preparation of probes for FISH

A probe for *A. cristatum* tandem repeat ACRI_CL78 (Said et al. 2018) was labeled by PCR with digoxigenin-dUTP (Roche, Mannheim, Germany) using diploid *A. cristatum* cv. Parkway DNA as a template. Biotin-dUTP (Roche) or digoxigenin-dUTP labeled probe for 5S rDNA was prepared according to Fukui et al. (1994) using rice DNA as a template for PCR. The plasmid pTa71 (45S rDNA) containing a 9-kb fragment from bread wheat with 18S-5.8S-26S rDNA and intergenic spacers (Gerlach and Bedbrook 1979) and genomic DNA of tetraploid *A. cristatum* PI 222957 were labeled with either biotin or digoxigenin by nick translation using standard kits (Nick Translation Mix, Roche) following the manufacturer’s instructions.

### Mitotic chromosome preparation

Seeds were germinated on moistened filter paper in a glass Petri dish in the dark at 25 °C for 3–4 days. Root tips were transferred to distilled water and incubated overnight at 1 °C in a box filled with ice water. Subsequently, the root tips were fixed in ice-cold 90% acetic acid for 10 min followed by three washes in 70% ethanol and stored in 70% ethanol at − 20 °C. Chromosome preparations were prepared using the drop technique according to Kato et al. (2004, 2006), with minor modifications as described in Danilova et al. (2012).

### Fluorescence in situ hybridization

Labeled probes for FISH and GISH were localized following the protocols of Cabrera et al. (2002) and Said et al. (2018) with modifications. Briefly, digoxigenin-labeled probes were detected using anti-digoxigenin fluorescein isothiocyanate (Roche). Biotin-labeled probes were detected with Cy3-conjugated streptavidin (Invitrogen, Life Technologies, Carlsbad, USA). The hybridization mixture (total volume = 10 μl/slide) contained 50 ng labeled probe DNA, 50% v/v formamide, 2 × SSC (0.15 mol/l NaCl plus 0.015 mol/l sodium citrate), 10% w/v dextran sulfate, 0.4 μg salmon sperm DNA and 0.1% w/v sodium dodecyl sulfate. In the case of GISH, 5 μg wheat genomic DNA was included in the hybridization mix as blocking DNA. The chromosomes and probes were denatured together at 80 °C for 3 min under high moisture conditions. The hybridization was carried out overnight at 37 °C. The slides were washed, the hybridization sites were detected, and chromosomes were mounted and counterstained with 4′,6-diamidino-2-phenylindole (DAPI) in Vectashield media (Vector Laboratories, Burlingame, USA).

### Microscopy, software, signal capture and image analysis

Chromosome preparations were examined using an Axio Imager Z.2 Zeiss microscope (Zeiss, Oberkochen, Germany) equipped with a Cool Cube 1 (MetaSystems, Altlussheim, Germany) camera and appropriate optical filter sets. The signal capture and image processing were performed using ISIS software (MetaSystems). The final image adjustment was done in Adobe Photoshop CS5 (Adobe Systems Incorporated, San Jose, USA).

### COS marker analysis

Genomic DNA was extracted from young leaves of wheat–*A. cristatum* chromosome and chromosome arm addition and translocation lines, from the diploid and tetraploid *A. cristatum* accessions and from bread wheat cv. Chinese Spring, using a QuickGene Mini 80 (FujiFilm, Tokyo, Japan) with a QuickGene DNA tissue kit (FujiFilm) according to the manufacturer’s instructions and was used as a template for PCR. Primers for 328 COS markers covering wheat homeologous groups 1–7 (group-1: 76, group-2: 16, group-3: 23, group-4: 120, group-5: 65, group-6: 15 and group-7: 13) were chosen from publicly available marker collections (Quraishi et al. 2009; Howard et al. 2011). Primer sequences for these markers and annealing temperature (Ta) are summarized in Supplementary Data S1. PCR was performed in 12 µl reaction volumes as described by Molnár et al. (2014, 2016) using a touchdown reaction profile: 94 °C 2 min, 10 cycles of 94 °C 0.5 min, Ta + 5 °C 0.5 min and decreased by 0.5 °C increments for every subsequent set of cycles, 72 °C 1 min, 30 cycles of 94 °C 0.5 min, Ta °C 0.5 min, 72 °C 1 min, hold at 72 °C 2 min in an Eppendorf Mastercycler (Eppendorf, Hamburg, Germany). PCR products of the 112 markers, which were analyzed in the Agricultural Institute, Hungarian Academy of Sciences (Martonvásár, Hungary), were separated by a Fragment Analyzer Automated CE System equipped with a 96-Capillary Array Cartridge with an effective length of 33 cm (Advanced Analytical Technologies, Ames, USA), and the results were analyzed and visualized by PROsize v2.0 (Advanced Analytical Technologies). The products of the remaining 216 markers, which were analyzed at the University of Córdoba, were separated on 2.5% agarose gels along with the O’RangeRuler™ 50 bp DNA size marker (Fermentas, Vilnius, Lithuania) as described by Nagy et al. (2006). The patterns were documented and analyzed using a GeneGenius gel documentation system (Syngene, Cambridge, UK). The PCR products were considered as polymorphic if difference in the product size between wheat and *Agropyron* was ≥ 3 bp.

### Sequence analysis

The orthologous relationship between the chromosomes of wheat and *A. cristatum* has been investigated from the genomic perspective of wheat. As a first step for the construction of the physical maps, the EST source sequences (Supplementary Data S2) were used as queries in BLASTn searches against the wheat reference pseudomolecules (Consortium (IWGSC) et al. 2018) to identify the start positions (bp) of the ESTs. Throughout the study, BLAST hits with *E* values smaller than 2.8e^−08^, identity % > 58.44 and alignment length > 100 bp were considered significant. The genomic start positions in bp of the best hits in wheat pseudomolecules (Supplementary Data S3) were used to construct physical maps of the polymorphic COS markers. The centromere positions for each wheat chromosome were obtained from the wheat reference genome sequence (Consortium (IWGSC) et al. 2018). The length in bp of wheat pseudomolecules, as well as the start genomic positions of the ESTs, was converted to pixels. Then, the BLASTn searches data were used to construct a physical map for each of the wheat chromosomes showing the position of the source EST of the COS markers assigned to *Agropyron* chromosomes. The wheat chromosomes were colored with six different colors representing the homeologous groups (1–6) of both species: wheat and *A. cristatum*. However, the marker name background was colored with colors correspond to their arm location on *A. cristatum* chromosomes as described by Molnár et al. (2016). Dark and light colors of the chromosomes and the background color of the marker name represent the short and long arms of wheat chromosomes and the arm location on *A. cristatum* chromosomes, respectively. The physical maps of the COS markers were designed using custom-made software.

## Accession numbers

Plant material; diploid *A. cristatum* cv. Parkway (2*n* = 2*x* = 14, PP), accession number PI 415799 and tetraploid *A. cristatum* (2*n* = 4*x* = 28, PPPP) accession PI 222957 are available at the USDA genebank (https://npgsweb.ars-grin.gov/gringlobal/search.aspx). Wheat–*A. cristatum* chromosome additions (1P, 2P, 3P, 4P, 5P and 6P) and telosome additions (2PS, 2PL, 3PS, 4PS, 5PL, 6PS and 6PL) as well as a wheat–*A. cristatum* 1PS·1BL translocation line are available upon request: Mahmoud Said (said@ueb.cas.cz), Institute of Experimental Botany, Šlechtitelů 31, CZ-78371 Olomouc, Czech Republic.

## Results

### Karyotype stability of the wheat–*A. cristatum* chromosome addition and translocation lines

**Fig. 2:**
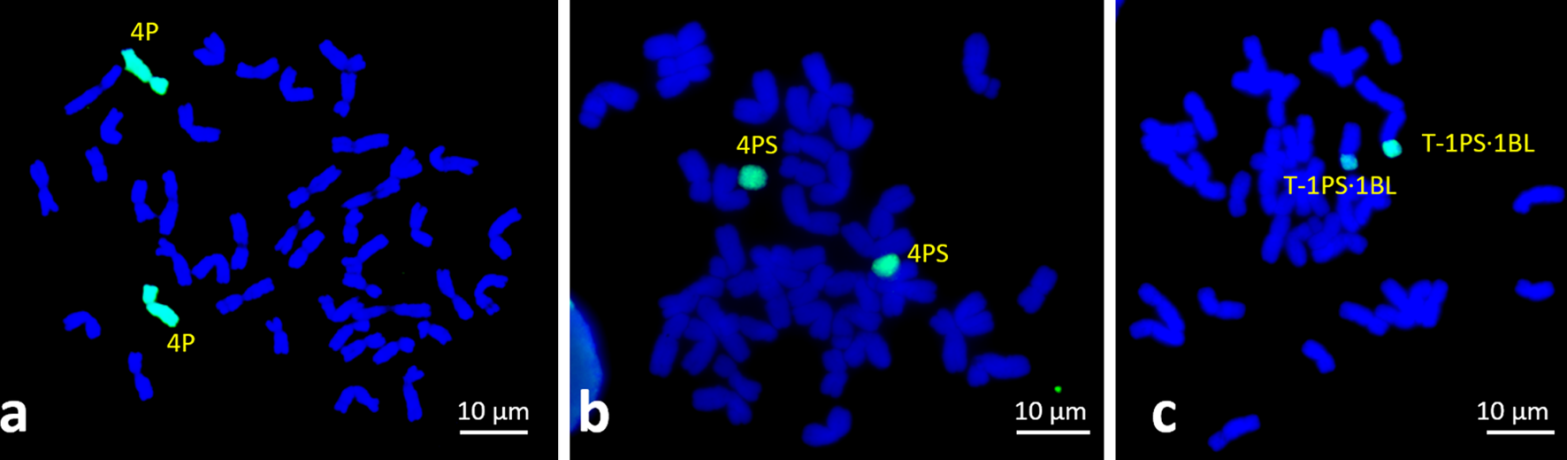
GISH on mitotic metaphase plates of wheat–*A. cristatum* lines using genomic DNA from tetraploid *A. cristatum* (green). **a** Chromosome 4P disomic addition **b** chromosome arm 4PS ditelosomic addition and **c** homozygous translocation 1PS·1BL lines. Chromosomes were counterstained by DAPI (blue). Please refer to the online version for the high-resolution color figure

Karyotype stability was observed in a majority of the addition and translocation lines (Supplementary Table S1). Complete stability was observed in CS-6PL addition lines and CST-P translocation lines with 100% maintenance of the disomic state of the alien chromatin. The second greatest stability was observed for addition lines CS-1P, CS-4P, CS-5P, CS-6P, CS-5PL and CS-6PS, where more than 90% of the progeny retained the disomic state. The CS-2P, CS-2PL, CS-3PS and CS-4PS addition lines were relatively stable, as more than 77% disomic progeny plants were identified. Furthermore, the CS-2PS addition line was unstable, with 47% ditelosomic and 10% monotelosomic progeny, while the remaining 43% plants did not retain any alien chromatin. Line CS-3P was the most unstable, as a high proportion of plants (74%) did not retain any alien chromatin, 17% of the progenies were monotelosomic, and 9% of the plants retained one chromosome 3P in addition to a chromosome arm later identified by FISH as 3PS. The 3PS chromosome arm was also detected in a new ditelosomic addition line CS-3PS, which was generated during the course of the study. This line showed high stability, where 77% of progenies maintained the ditelosomic state. Although the CS-6PS addition line was considered highly stable with 90% ditelosomic plants in the progeny, 5% of plants carried isochromosome 6PS and the remaining 5% were monotelosomic as revealed by GISH. The wheat–*A. cristatum* lines with disomic and ditelosomic additions confirmed by GISH (Fig. 2, Supplementary Fig S1) were selected for COS marker analyses.

We observed two karyotypes in the progenies of line CS-3P, one retaining one chromosome 3P and one arm 3P, and the other karyotype of a new line possessing two telosomes (Fig. 1). Based on the FISH pattern of the tandem repeat ACRI_CL78 (Said et al. 2018), the telosomes were identified as a homologous pair 3PS of a new ditelosomic addition line CS-3PS (Supplementary Fig S2). Furthermore, the telosomes of the new line CS-3PS had similar FISH pattern to the short arm of chromosome 3P of line CS-3P (Supplementary Fig S2). The results were also confirmed by the comparison of the FISH pattern of the tandem repeat on the short arm of chromosome 3P and the telosome in line CS-3P possessing one chromosome 3P and one arm (Supplementary Fig S2). The unidentified tetraploid *A. cristatum* chromosome short arm translocated to wheat chromosome arm 1BL (Ochoa et al. 2015) in the translocation line CST-P was identified in the present work by FISH as 1PS based on the molecular karyotype of *A. cristatum* (Said et al. 2018), as it is possible to distinguish between chromosomes 1P and 5P. Although both are characterized by 45S rDNA signals at the terminal position of the short arms, chromosome 5P has a 5S rDNA locus at the subterminal position of the short arm. This was also confirmed by comparing the distribution patterns of 45S rDNA on this chromosome arm in the translocation line with the patterns of 5S and 45S rDNA on tetraploid *A. cristatum* PI 222957 (Supplementary Fig S3), which was used by Ochoa et al. (2015) to develop the translocation. Consequently, based on these observations, the translocation line (CST-P) was renamed to CST-1PS·1BL.

The new wheat–*A. cristatum* CS-3PS ditelosomic line and the translocation line CST-1PS·1BL were also involved in the COS marker study (Fig. 2, Supplementary Fig S1). Because the whole 3P chromosome was not represented in the set of wheat–*A. cristatum* disomic addition lines, we used line CS-3P which possesses one chromosome 3P and one telosome 3PS (Supplementary Fig S1).

### Fertility and morphological traits

**Fig. 3:**
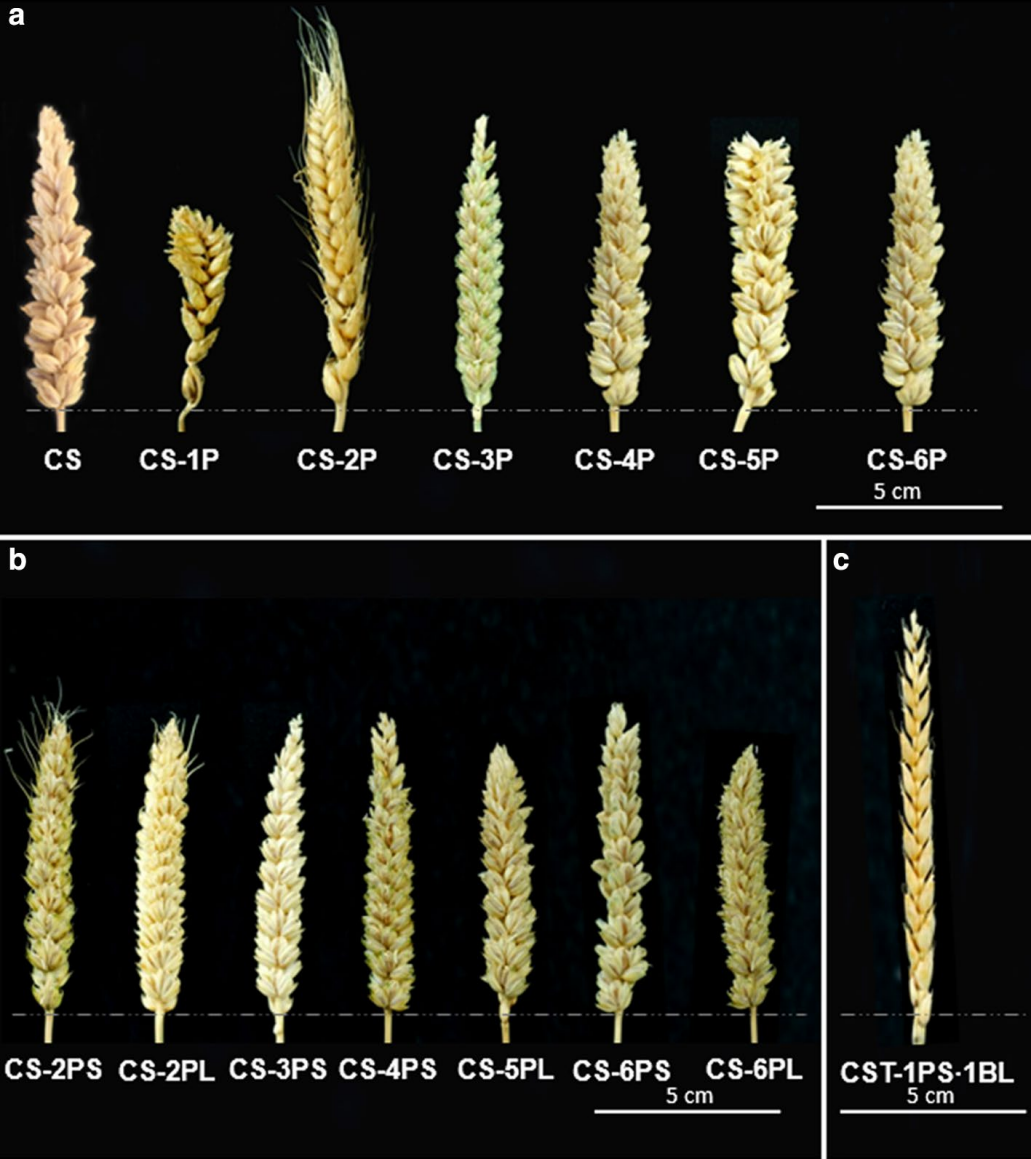
Spike morphology of wheat–*A. cristatum* chromosome (1P–6P) disomic addition lines (**a**), ditelosomic addition lines (**b**) and translocation 1PS·1BL (**c**) in CS

The observations on fertility and spike morphology are summarized in Supplementary Table S2 and Fig. 3. All lines were fertile and vigorous over the generations, both in a greenhouse and under field conditions. The lines differed in spike morphology in terms of color, size and shape, and statistically significant differences were found between the lines for the evaluated characters. In particular, translocation line CST-1PS·1BL had a significantly longer spikes compared to CS, while the CS-1P addition line had the shortest spike with approximately half the spike length of CS. Line CS-1P had significantly fewer spikelets per spike compared to the remaining lines, while CS-2PL showed the significantly highest value for this trait. With respect to seed number per spike, CS-6P had a greater mean value, but the difference was not significant when compared to CS, CS-4PS and CS-6PL. Line CS-2P had the lowest number of seeds per spike, but the difference was not significant compared to CS-1P and CS-4P. In this study, all wheat–*A. cristatum* lines yielded awnless spikes, except for line CS-2P which had awned spikes, and fewer and shorter awns were also observed on the upper spikelets of CS-2PS and CS-2PL (Fig. 3).

### Assignment of COS markers to P chromosomes

**Fig. 4:**
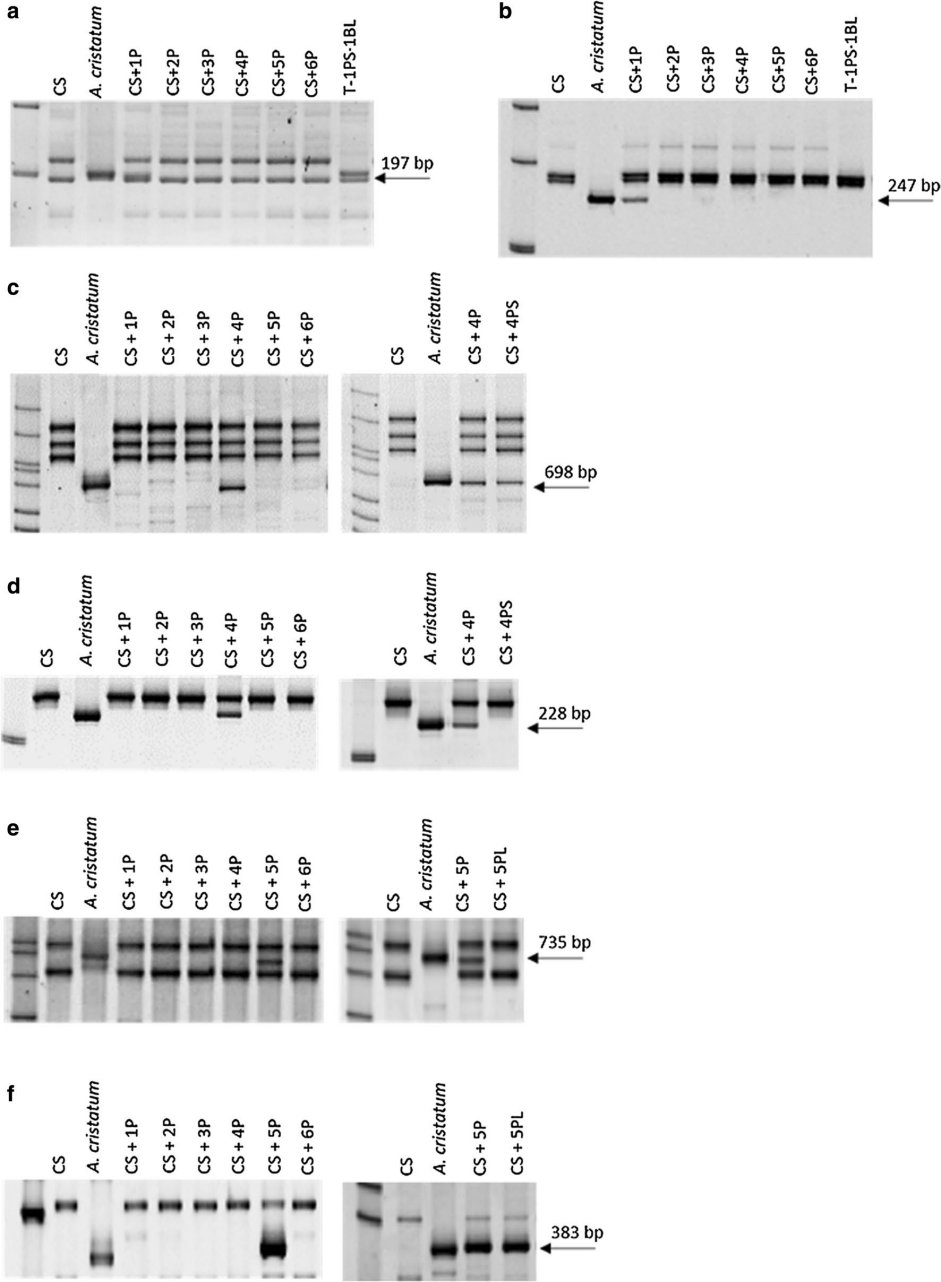
PCR amplification profiles used for the location of COS molecular markers on chromosomes 1P, 4P and 5P. **a**, **b** c737237 and c743346 mapped on the short and long arms of chromosome 1P, respectively; **c**, **d** c763059 and c739175 mapped on the short and long arms of chromosome 4P, respectively; **e**, **f** c728036 and c762245 mapped on the short and long arms of chromosome 5P, respectively

The confirmation of the presence of chromatin originating from tetraploid *A. cristatum* in wheat–*A. cristatum* addition and translocation lines made them suitable for the subsequent COS markers analysis. Out of the 328 markers tested for transferability to tetraploid *A. cristatum* (Table 1), 279 (85.1%) consistently amplified products in tetraploid *A. cristatum* PI 222957, and, out of these, 139 (49.8%) were polymorphic between tetraploid *A. cristatum* and wheat (CS) (Fig. 4 and Table 1). The highest level of polymorphism (90.0–90.9%) was observed for the markers of wheat chromosome groups 3 and 6, while the markers of wheat chromosome groups 1 and 4 showed a relatively low level of polymorphism (32.0–35.0%). Using wheat–*A. cristatum* disomic- and ditelosomic addition lines, sixty-nine out of the 139 polymorphic markers were assigned to the P genome chromosomes. Because some markers were assigned to more than one P chromosome (Table 2), the total number of markers (69) assigned to tetraploid *A. cristatum* chromosomes was different from the sum (78) of the specific markers per P chromosome (no. of markers/no. of PCR amplicons per chromosome: 1P: 11/15; 2P: 6/7; 3P: 10/14; 4P: 19/24; 5P: 21/24; 6P: 11/13).

**Table 1:** Number and frequency of wheat COS markers amplified in tetraploid *A. cristatum*

Wheat group	Polymorphic	Nonpolymorphic	Total number amplified
1	18 (32.7)^a^	37 (67.3)	55
2	11 (78.5)	3 (21.5)	14
3	18 (90.0)	2 (10.0)	20
4	42 (34.7)	67 (61.0)	109
5	27 (52.9)	24 (47.1)	51
6	10 (90.9)	1 (9.1)	11
7	9 (69.2)	4 (30.8)	13
uk^b^	4 (66.6)	2 (33.3)	6
Total	139 (49.8)	140 (50.2)	279

^a^Number in parenthesis are the percentages of wheat markers relative to the total number of amplified markers

^b^Unknown wheat group location in Howard et al. (2011)

**Table 2:** COS markers polymorphic between wheat and *A. cristatum* and their chromosomal location in wheat and in the P genome chromosomes

Marker	Product size in *T. aestivum*	Product size in *A. cristatum*^d^	Product size in *A. cristatum*^e^	Chr. location in *A. cristatum*	Chr. location in wheat^a^	Location in wheat^b^
c732651^cf^	250	–	225	1PS	1BS	1ABD
c744391^cf^	382	–	487	1PS	1AS-1BS	1ABD
c737237^cf^	230	–	197	1PS	1BS-1DS	1ABD
c740349^cf^	320	–	345	1PL	1AS	1ABD
c743346^cf^	278	–	247	1PL	1BS-1DS	1ABD
c744533^f^	241	244	256	1PL	1AL-1BL-1DL	1ABD
c737520^f^	368	416	416	1PS	1BS	1ABD
	372	467	467	1P		
	387	494	472	1P		
	436	–	–	–		
1J^f^	169	169	205	1PL	1ABD	1ABD
	–	182	227	1PL		
TR574^f^	241	239	245	1PL	1ABD	1ABD
	254	251	–	–		
	258	–	–	–		
TR451^i^	257	278	301	3PL	2ABD	–
	269	–	318	3PL		
2R^g^	282	297	–	2PL, 5PL	2ABD	2ABD
	291	–	–	–		
2N^g^	564	579	560	5PL, 6PS	2ABD	2ABD
	571	–	585	2PL		
	589	–	–	–		
c744070^g^	221	231	231	2PL	2AL-2BL-2DL	2ABD
	226	–	234	6PS		
	255	–	–	–		
c746642^f^	661	674	1032	2PL	2AL-2BL-2DL	2ABD
	–	718	1190	2PL		
TR430^i^	268	264	315	2PL	2ABD	–
TR390^h^	226	254	217	1PS	2ABD	3ABD 5ABD
	230	263	222	1P		
c755442^f^	821	708	773	3PS	3AS-3BS-3DS	3ABD
	841	745	–	–		
TR63^f^	439	450	459	3PS	3ABD	3ABD
	455	479	–	–		
c803223^g^	462	–	471	1P, 3PL, 4PL, 6PL	3AL-3BL-3DL	3ABD
	475	–	–	–		
	489	–	–	–		
TR72^f^	170	264	263	3PS	3ABD	2ABD
	203	276	274	3PS		
	239	–	–	–		
TR60^f^	241	169	169	3PL	3ABD	3ABD
TR73^i^	522	446	493	3P	3ABD	3ABD
c767527^f^	327	338	366	3PS	3AL-3BL-3DL	3ABD
	342	–	412	3PS		
TR85^i^	218	210	245	3P	3ABD	3ABD
	225	235	–	–		
	–	242	–	–		
TR471^h^	268	283	329	3PS		5ABD
	279	292	339	3PS		
	290	–	–	–		
BE404977^cf^	262	–	320	4PL	4AL-4BS-4DS	4ABD
c737898^cf^	439	–	560	4PS	4AS-4BL-4DL	4ABD
c738073^cf^	275	–	210	4PL	4BS-4DS	4ABD
c739175^cf^	251	–	228	4PL	4AL-4DS	4ABD
c740051^cf^	871	–	950	4PS	4AL-4B-4D	4ABD
c740946^cf^	385	–	295	4PL	4BS-4DS	4ABD
c750766^i^	744	–	800	4PS	4AS-4BL-4DL	4ABD
c756425^ci^	251	–	198	4PS	4AL-4DL	4ABD
c757404^cf^	592	–	610	4PS	4AS-4BL-4DL	1ABD 4ABD
c760004^cf^	781	–	908	4PL	4AL-4BS-4DS	4ABD
c763059^cf^	877	–	698	4PS	4DL	4ABD
c769630^cf^	258	–	350	4PS	4AS-4BL-4DL	4ABD
c797119^ch^	474	–	400	6PL	4AS-4BL-4DL	4ABD
c765452^f^	321	312	345	4PS	4AS-4BL-4DL	4ABD
	329	366	354	4PS		
	–	–	389	4PS		
TR636^f^	272	233	233	2PL	4ABD	2ABD 6ABD
	–	251	–	–		
	–	281	–	–		
c771467^f^	287	287	294	4PL	4AL-4BS-4DS	4ABD
TR118^h^	–	–	377	4PL		
	493	591	591	5PL	4ABD	4ABD
TR647^f^	311	289	298	4PS	4ABD	4ABD
	316	298	–	–		
	330	–	–	–		
c771529^f^	353	224	377	4PS	4AS-4BL-4DL	4ABD
	362	369	382	4PS		
	–	–	408	4PS		
TR133^h^	689	718	707	5PL	4ABD	4ABD
c728036^cf^	624	–	735	5PS	5BS	5ABD
c739859^cf^	528	–	451	5PS	5AS-5BS	5ABD
c746156^ci^	338	–	375	5PS	5AS-5BS	5ABD
c762599^cf^	295	–	306	5PS	5AS-5BS-5DS	5ABD
c796275^cf^	461	–	489	5PS	5AS-5BS	5ABD
c745899^cf^	644	–	695	5PL	5AL-5BL-5DL	5ABD
c748166^cf^	266	–	212	5PL	5AL-5BL-5DL	5ABD
c750235^cf^	277	–	330	5PL	5AL-5BL-5DL	5ABD
c756721^cf^	301	–	282	5PS	5AL-5BL-5DL	5ABD
c759134^ci^	735	–	796	5PS	5BL-5DL	5AD
c762245^cf^	399	–	383	5PL	5BL	5ABD
TR764^i^	204	230	230	5PL	5ABD	5ABD
	214	239	239	5PL		
TR128^f^	211	218	218	5PL	5ABD	5ABD
	261	–	–	–		
c749645^f^	345	126	395	5PL	5AL-5BL-5DL	5ABD
	363	338	406	5PL		
TR759^h^	335	335	340	6PL	5ABD	5ABD
	–	340	–	–		
TR766^f^	280	270	449	5PL	5ABD	5ABD
c717465^f^	261	258	258	5PL	5BL-5DL	5ABD
	–	273	273	5PL		
TR4^h^	245	295	272	5PL		3ABD
	289	–	–	–		
c747871^f^	736	744	740	6PS	6AS-6BS-6DS	6ABD
	–	840	–	–		
c724406^f^	630	574	650	6PS	6BL-6DL	6ABD
	–	630	667	6P		
BE445667^f^	248	208	268	6PL	6ABD	6ABD
	–	223	–	–		
BE490226^f^	460	442	464	6PL	6ABD	6ABD
	517	452	503	6PL		
BE426214^g^	319	348	304	4PL	6ABD	6ABD
	327	356	304	6PS		
	334	377	–	–		
	350	–	–	–		
	360	–	–	–		
TR88^f^	416	420	420	6PS		6ABD
	–	492	–	–		

The 1PS·1BL translocation line (Ochoa et al. 2015) was used for the localization of COS markers to the short arm of chromosome 1P from tetraploid *A. cristatum* accession PI 222957

^a^Quraishi et al. (2009) and Howard et al. (2011)

^b^Determined by BLASTn using source ESTs of the markers as queries to the reference sequences of bread wheat chromosomes, the centromere positions for each wheat chromosome were determined from Consortium (IWGSC) et al. (2018)

^c^Markers tested in University of Córdoba

^d^Determined in the tetraploid *A. cristatum* accession PI 222957

^e^Determined in the wheat–*A. cristatum* chromosome addition lines and ditelosomic lines, where the accessions of *A. cristatum* parental line are unknown

^f^Markers located on the same homeologous chromosome group in tetraploid *Agropyron* as in wheat

^g^Markers located on homeologous and nonhomeologous tetraploid *A. cristatum* chromosomes

^h^Markers assigned to nonhomeologous P genome chromosomes

^i^Markers excluded from wheat–*A. cristatum* homeologous relationships, their source ESTs BLASTed to the sequences of the wheat chromosomes gave no hits, or the alignment length was below the threshold (100 bp)

The availability of CS-*A. cristatum* ditelosomic lines provided the opportunity to locate COS markers on chromosome arms. Because ditelosomic lines for the short and long arms were available only for chromosomes 2P and 6P, only one of the two arms could be checked for chromosomes 3P, 4P and 5P and for 1P using the CST-1PS·1BL translocation. Therefore, if the PCR results were negative in the available telosomic line and positive in the whole chromosome addition line, we concluded that the COS marker was located on the opposite arm. In this manner, sixty-seven out of the sixty-nine markers were assigned to chromosome arms of the tetraploid *A. cristatum* (Table 2). We failed to map the remaining two markers to particular chromosome arms. The tetraploid *Agropyron* chromosome-specific markers showed a significant level of length polymorphism (3–558 bp, mean 54.59 bp) between wheat and the parental tetraploid *A. cristatum* genotype represented by the wheat–*A. cristatum* addition and ditelosomic lines. Therefore, they were considered suitable for marker-assisted selection of new wheat–*Agropyron* introgression lines in prebreeding programs. In this study, 90 polymorphic loci of 69 markers (1–3 loci, 1.30 loci per marker) covering from one to six homeologous groups of the P genome were found to be suitable for high-throughput detection of tetraploid *A. cristatum* chromosomes.

### Wheat–*A. cristatum* homeology at the chromosome level

**Fig. 5:**
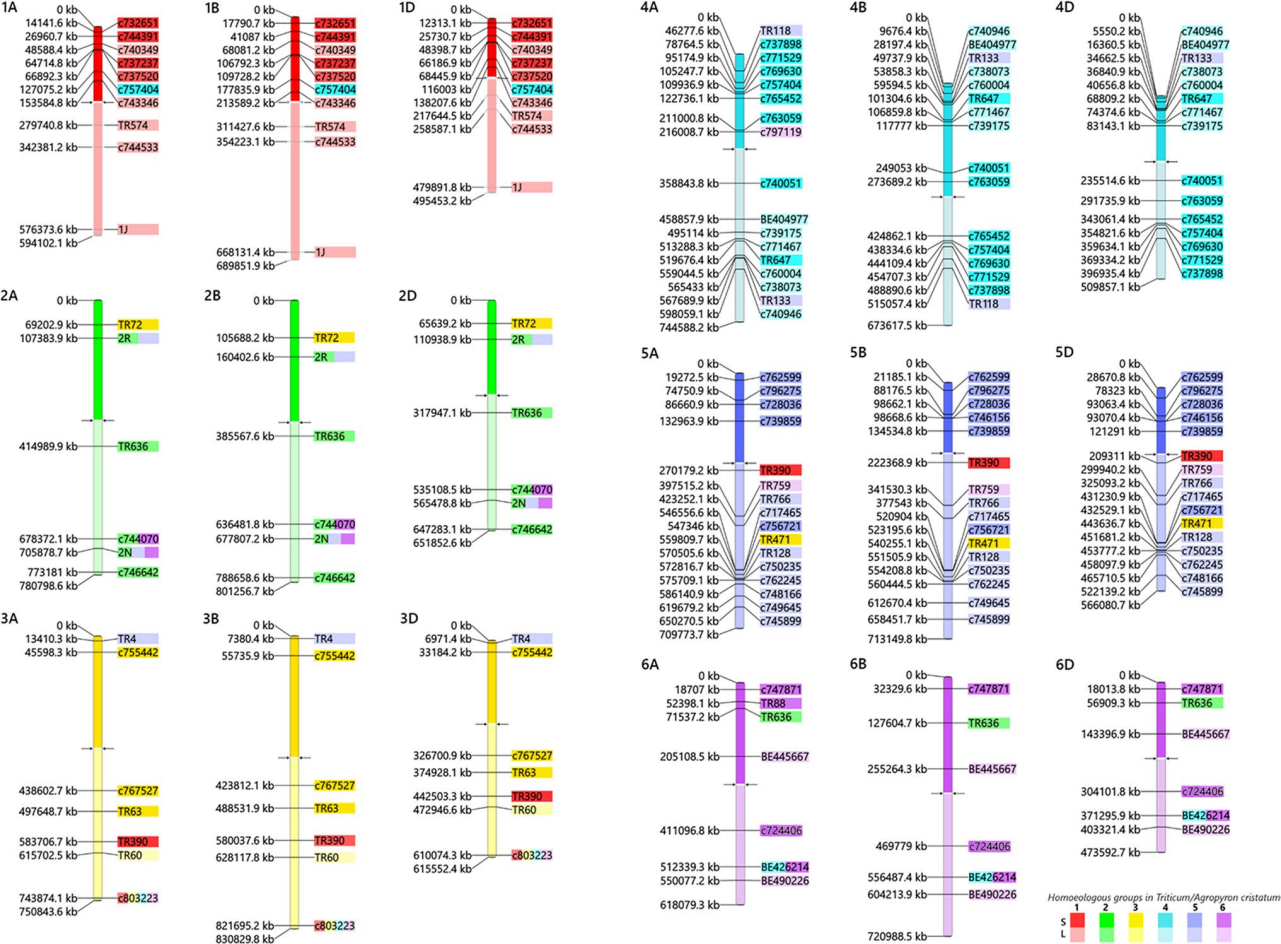
Genome relationships defined by polymorphic COS markers in wheat and *A. cristatum* from the genomic perspective of wheat homeologous groups 1–6 (Group-7 was omitted from this study due to the unavailability of a wheat–*A. cristatum* 7P addition line). The name of the markers is on the right, while their physical positions (kb) on the wheat pseudomolecules are on the left. The wheat chromosomes were colored with six different colors representing homeologous groups (1–6). The background color of the marker name indicates the chromosomal location of the marker in *A. cristatum*. If the chromosome and the background of the marker name have the same color, the marker assigned to the same homeologous group chromosome in wheat and *A. cristatum*. For example, the group 1 chromosomes of wheat are red and the background of the nine marker names is red, indicating that these nine markers are assigned to the group 1 chromosomes in wheat and *Agropyron* (1ABD and 1P). However, the turquoise color indicates that the marker c757404, which is located on the 1A, 1B and 1D at 127075.2, 177835.9 and 116003 kb, respectively, in wheat, was assigned to 4P chromosome of *A. cristatum*. Dark and light colors indicate short (S) and long (L) arms, respectively. The centromere positions (arrows) for each wheat chromosome were determined from the wheat reference genome sequence (Consortium (IWGSC) et al. 2018). Please refer to the online version for higher resolution color figure

To investigate wheat–*A. cristatum* homeologous relationships at the chromosome level, the source ESTs of the 69 polymorphic COS markers were BLASTed to the sequences of the wheat chromosomes (Consortium (IWGSC) et al. 2018). Sixty-seven markers showed hits on wheat pseudomolecules, and two markers (TR451, TR430) gave no hits. Seven markers (TR73, TR85, c750766, c756425, c746156, c759134 and TR764) were excluded from the subsequent analysis because the alignment length was below the threshold (100 bp). For the remaining markers, the start positions of the alignments of the best hits on the A, B and D genomes were extracted to produce a physical map from the perspective of the wheat genome (Supplementary data S3). In the map, the chromosomal locations of the markers in tetraploid *A. cristatum* were visualized by different colors of the marker name background, which provides an overview of the wheat–*A. cristatum* genome relationships from the perspective of the wheat genome (Fig. 5). In the physical map, the coverage of wheat chromosome groups 2, 3 and 6 with COS markers was smaller (6–7 markers per chromosome group) than wheat chromosome group-1 (10 markers) and wheat chromosome groups 4 and 5 (15–16 markers per chromosome).

Most of the markers (48) located on the same **h**omeologous (H) chromosome group in tetraploid *Agropyron* as in wheat, while seven markers were assigned to **n**onhomeologous (N) P genome chromosomes. The remaining five markers have duplicated loci as they were assigned to both **h**omeologous and **n**onhomeologous (H/N) chromosomes of *A. cristatum*.

The marker c757404, which is located on the interstitial part of the short arm of wheat chromosome group-1, was assigned to the short arm of 4P. The marker TR72, specific to the subtelomeric region of wheat chromosome group-2 short arms, was located at 3P. The marker TR4, specific to the subtelomeric region of short arms of wheat chromosome group-3, was assigned to 5PL, while TR390, specific to the long arms of the interstitial part of wheat chromosome group-3, was located at 1P. On wheat chromosome group-4, two markers (TR118 and TR133) were mapped to the 5P chromosome and one marker c797119 was assigned to 6P. The wheat chromosome group-5 markers TR390, TR759 and TR471 were detected on chromosomes 1P, 6P and 3P, respectively, while the duplicated locus TR636, which is located on wheat chromosome groups 2 and 6, was identified on 2P.

### Inter- and intrachromosomal rearrangements in the P genome

As shown in Table 2 and in the physical map (Fig. 5), 53 (48 H and 5 H/N) out of 60 (88.3%) COS markers showed synteny between the bread wheat and tetraploid *A. cristatum* genomes as they were detected on the same homeologous chromosome groups, with 46 (76.7%) of them at the same short or long arm in tetraploid *Agropyron* and wheat. However, the remaining seven (11.7%) COS markers mapped to nonhomeologous chromosomes and thus revealed structural differences between the chromosomes. Ten markers (16.7%) indicated intrachromosomal rearrangements as these markers were specific for the same homeologous chromosomes in tetraploid *Agropyron* as in wheat, but they located at the opposite arms. These kinds of intrachromosomal rearrangements were found on *A. cristatum* chromosomes 1P, 3P, 4P, 5P and 6P (Supplementary Table S3 and Fig. 5). Furthermore, the chromosomal location of other COS markers revealed some intragenomic duplications in tetraploid *A. cristatum* relative to wheat (Table 2 and Fig. 5). Three duplications were detected by markers specific to wheat chromosome group-2. Loci for marker 2R on the short arms of wheat chromosome group-2 detected a duplication of 2PL/5PL, while markers from long arms detected two duplications 2PL/6PS and 2PL/5PL/6PS were found by the markers c744070 and 2N, respectively. One duplication, 1PL/3PL/4PL/6PL, was detected by the marker c803223, which is specific for the telomeric region of wheat chromosome group-3, while a 4PL/6PS duplication was found by the marker BE426214, which is specific for the long arms of wheat chromosome group-6.

## Discussion

The present study significantly increased the number of chromosome-specific molecular markers suitable for improving the selection throughput of wheat–*A. cristatum* introgression lines with requested alien chromatin.

Before using the wheat–*Agropyron* lines for structural genomic studies, cytogenetic control is needed because the elimination of alien chromosomes from wheat–alien genetic stocks is a well-known feature (Taketa et al. 1995; Molnár-Láng et al. 2005; Szakács and Molnár-Láng 2010). In this work, different levels of instability were observed in wheat–*A. cristatum* lines, particularly in CS-3P addition line, where the *Agropyron* chromosome was not observed in disomic form. These results are in line with previous studies demonstrating that wheat cytogenetic stocks carrying chromosome 3 from wild relatives of wheat are difficult to maintain in disomic form (Miller et al. 1982; Said et al. 2012). A recent study using 3D-FISH on wheat–rye introgression lines showed that incorrect arrangement in the nuclei at the beginning of meiosis may prevent the migration of alien introgressed chromosomes into the telomere bouquet, thereby preventing synapsis and chiasma formation, and leading to their gradual elimination (Perničková et al. 2019). A similar study in the future may help to understand the cytogenetic background of the high eliminating rate of 3P chromosomes from the wheat–*A. cristatum* addition line.

The cytogenetic analysis allowed to identify a new 3PS ditelosomic addition line using the tandem repeat probe ACRI_CL78 which has a subterminal signal diagnostic for 3PS (Said et al. 2018). By the use of 5S rDNA and 45S rDNA probes, we also identified the P genome chromosome arm involved in the wheat–*A. cristatum* centric fusion CST-P·1BL developed by Ochoa et al. (2015) as 1PS according to the *A. cristatum* karyotype developed by Said et al. (2018). The identification of *A. cristatum* chromatin in these two lines enabled the assignment of markers to 1PS and 3PS.

Spike architecture and fertility are important components contributing to the yield of bread wheat. Our results indicating that chromosomes 2P, 4P and 6P carrying genes contributing positively to the number of spikelets per spike which is accompanied by a reasonable fertility (2.76–2.81) in CS-6P, CS-6PL and CS-4PS resulting in the same level of seeds per spike as the parental wheat genotype CS. These results support the previous observations on the positive effect of chromosome 6P on grain yield of bread wheat reported by Wu et al. (2006), Ye et al. (2015) and Zhang et al. (2015). The effect of *Agropyron* genes on the spike architecture of wheat might be more pronounced after shortening the size of introgressed 4P or 6P chromatin by induced homeologous recombination or by random chromosome breakage (Shi et al. 2005; Endo 2015; Martín et al. 2017). A marker-based system would also make more effective the selection of genotypes with shorter chromatin.

Easy to use, chromosome-specific molecular markers are a prerequisite for increasing the selection throughput of wheat–alien introgression lines with desirable karyotypes. The co-dominant COS markers meet this criteria, as they amplify specific PCR products from wheat and the introgressed chromatin is suitable for discrimination between heterozygotes and homozygotes in F2 and BC populations. One of the two primers is specific for the conserved exon sequences; thus, the markers have theoretically high transferability between the species, while the other primer is positioned, so that the product covers the intron. As intron sequences are less conserved than exons, the size-polymorphic product obtained in wheat–alien introgression lines reflects the species specific genetic variations relative to wheat.

We observed 85.1% transferability of COS markers between wheat and tetraploid *A. cristatum*, which is less than those reported by Linc et al. (2017), who found 92.1% transferability of COS markers between wheat and diploid *A. cristatum*. However, it is higher than those found by Copete and Cabrera (2017), who obtained 68.2% transferability of wheat chromosome groups 2 and 6 specific COS markers between wheat and tetraploid *A. cristatum*. In comparison with other marker systems, the transferability of wheat-specific COS markers can be higher than EST-STS markers, it can be comparable with those of EST-SSRs and can be lower than those of PLUG markers as it was reported for *Aegilops caudata* (Gong et al. 2017) and *Aegilops searsii* (Gong et al. 2016).

In this work, we significantly increased the number of PCR-based markers available for detection of chromosomes 1P–6P of tetraploid *A. cristatum* and their arms in the wheat background. We found that out of the 279 COS markers producing amplicons in *A. cristatum*, 139 (49.8%) showed size-polymorphic product between wheat and tetraploid *A. cristatum*. A similar range of size polymorphism (54.27%) was reported by Han et al. (2014) for EST-SSR markers between the wheat cultivar ‘Fukuhokomugi’ and tetraploid *A. cristatum* genotype Z559. Interestingly, the same work showed a much smaller percentage of size polymorphism (36.95%) for genomic SSR markers. Our work significantly expanded the number of P chromosome-specific markers by identifying the chromosomal locations of 69 COS markers covering the 1P–6P chromosomes of tetraploid *A. cristatum*. These polymorphic markers are considered potentially useful to follow tetraploid *A. cristatum* chromosomes in bread wheat backgrounds during prebreeding programs.

Tetraploid *A. cristatum* is considered autopolyploid, and the markers are specific for its both subgenomes. However, intraspecific diversity may cause presence/absence or size variation in the amplified loci. In a previous work, Linc et al. (2017) investigated genetic diversity using COS markers in perennial grass species, including two accessions of diploid *A. cristatum*. Among the 96 markers gave amplicon on *A. cristatum*, 46 (76 loci) were nonpolymorphic, while 50 markers (106 loci) were polymorphic between the two accessions of *A. cristatum*.

Homeologous relationships between wheat and related species provide important information for the targeted development of markers specific to alien chromosome regions potentially responsible for important agronomic traits as demonstrated for *Aegilops ventricosa* (Burt and Nicholson 2011). The present study revealed close homeologous relationships between the chromosome arms of bread wheat and tetraploid *A. cristatum* as 48 markers were located on the same homeologous chromosomes of wheat as those of *Agropyron*. However, this arm-level homeology was perturbed in some loci as one and one markers indicated 1ABDS-4P, 2ABDS-3P, 3ABDS-5P, 3ABDL-1P, 4ABD-5P, 4ABD-6P, 5ABD-1P, 5ABD-3P, 5ABD-6P and 6ABDS-2P relationships, while others indicated 2P/5P or 2P/5P/6P duplications. These results agree well with those of Zhou et al. (2018) who used wheat 660 k SNP array to genotype a diploid *A. cristatum* × *Allium mongolicum* segregating population and also found the P genome of *Agropyron* is generally collinear, but some rearrangements exist relative to wheat genomes. Some markers are also indicated intrachromosomal rearrangements, such as peri- and paracentric inversions, on the P genome chromosomes, which is in line with the cytogenetic observations of Said et al. (2018), who used single-gene FISH and detected rearrangements on chromosomes 2P–7P, including peri- and paracentric inversions on 4P and 6PL, respectively, of diploid *A. cristatum* relative to wheat. Our results based on the chromosomal location of COS markers may indicate that chromosomal inversions are more abundant in the P genome of tetraploid *A. cristatum* than those of the diploid form, which could be the consequence of the polyploidization-induced genome reorganization (Ma et al. 2004; Han et al. 2005, 2017; Zhang et al. 2013). In line with these studies, Said et al. (2019) observed differences in the FISH signal pattern between chromosomes flow sorted from the diploid and tetraploid accessions, and the differences were more pronounced on the chromosomes flow sorted from tetraploid *A. cristatum*. A recent development in the flow-cytometric sorting of P genome chromosomes from the wheat–*A. cristatum* addition and ditelosomic lines opens the way for shot gun sequencing and high-resolution structure analysis of *A. cristatum* chromosomes (Said et al. 2019).

## Conclusions

In the present study, a set of COS markers was successfully assigned to the chromosomes and chromosome arms of the P genome of tetraploid *A. cristatum*, which is the only form of this wild species suitable for chromosome-mediated gene transfer to bread wheat. Our results revealed the genome structure and the homeologous relationships of this species relative to wheat, which could help us to understand the evolution of species from the Triticeae tribe, open the door for genome analysis and support the use of this important wild gene source in wheat breeding.

### Author contribution statement

AC, JD, JV, IM and MS conceived the project. ACP, EG, JV, IM and MS performed the experiments and drafted the manuscript; all authors contributed to the manuscript writing and approved the final version.

## Electronic supplementary material

Below is the link to the electronic supplementary material.

Supplementary material 1 (DOC 1664 kb)

Supplementary material 2 (DOC 93 kb)

Supplementary material 3 (XLSX 31 kb)

Supplementary material 4 (XLSX 25 kb)

Supplementary material 5 (XLSX 25 kb)
