# Supplementary material for: Uncovering homeologous relationships between tetraploid Agropyron cristatum and bread wheat genomes using COS markers
Source: Theor Appl Genet. 2019 Jul 16;132(10):2881–98. doi: 10.1007/s00122-019-03394-1 (PMC6763527; doi:10.1007/s00122-019-03394-1)
Supplement: Supplementary file 1 — Supplementary material 1 (DOC 1664 kb) [file 122_2019_3394_MOESM1_ESM.doc]

**Uncovering homoeologous relationships between tetraploid *Agropyron cristatum* and bread wheat genomes using COS markers**

**Mahmoud Said^1,2^, Alejandro Copete Parada^3^, Eszter Gaál^4^, István Molnár^1,4^ Adoración Cabrera^3^, Jaroslav Doležel^1^, Jan Vrána^1*^**

^1^Institute of Experimental Botany, Center of the Region Haná for Biotechnological and Agricultural Research, Šlechtitelů 31, CZ-78371 Olomouc, Czech Republic

^2^Field Crops Research Institute, Agricultural Research Centre, 9 Gamma Street, Giza, 12619 Cairo, Egypt

^3^Genetics Department, ETSIAM, Agrifood Campus of International Excellence (ceiA3), University of Córdoba, 14071, Spain

^4^Agricultural Institute, Centre for Agricultural Research, Hungarian Academy of Sciences, Martonvásár, Hungary

*Jan Vrána

Email: [vrana@ueb.cas.cz](mailto:vrana@ueb.cas.cz)

Tel: +420 585 238 720

**
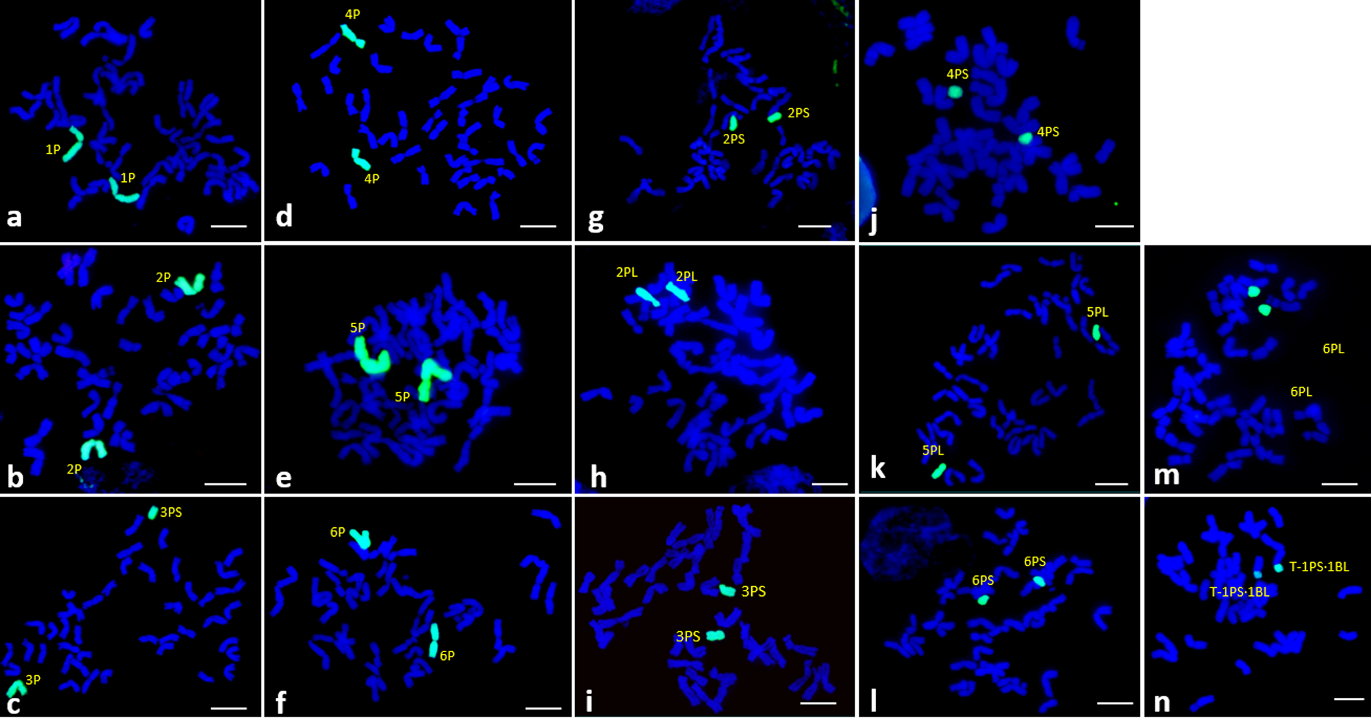
**

**Supplementary Fig. S1** GISH on mitotic metaphase plates in wheat-*A. cristatum* chromosome addition lines (a-f), ditelosomic addition lines (g-m) and homozygous translocation line 1PS·1BL (n) using genomic DNA from *A. cristatum* (green). The chromosomes were stained by DAPI (bue). Bars = 10 μm

**
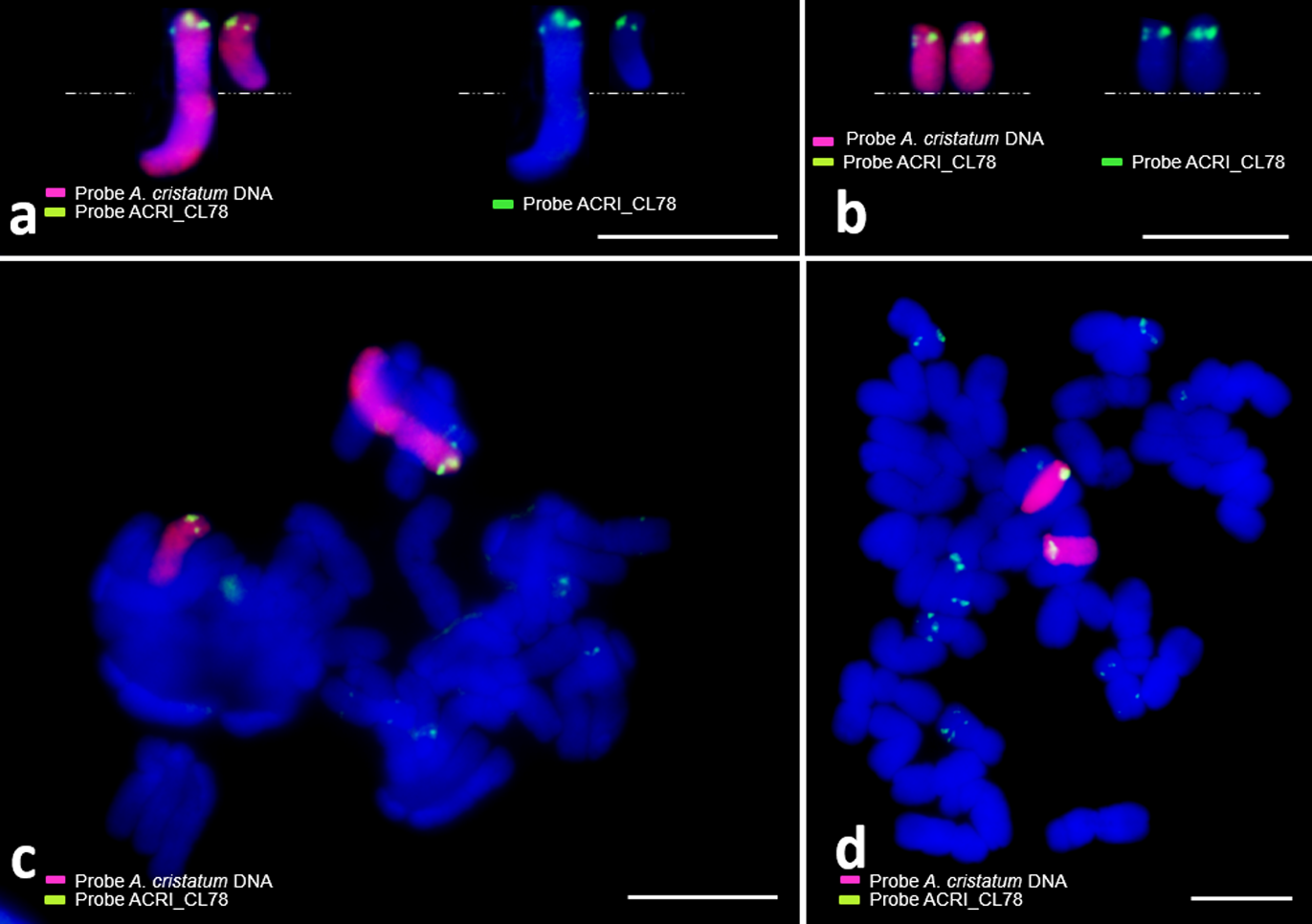
**

**Supplementary Fig. S2** FISH and GISH on chromosomes 3P and 3PS in the genetic background of wheat. **a**) The FISH pattern of the probe for ACRI_CL78 repeat (green) on chromosome 3P (left) and 3PS (right), in wheat-*A. cristatum* addition line monosomic 3P and monotelosomic 3PS. **b**) The FISH pattern of probe ACRI_CL78 (green) on 3PS in wheat-*A. cristatum* 3PS ditelosomic addition line. **c**) GISH using DNA from *A. cristatum* (red) and FISH with a probe for ACRI_CL78 (green) on chromosome 3P and 3PS in the genetic background of wheat. **d**) GISH using DNA from *A. cristatum* (red) and FISH with a probe for ACRI_CL78 (green) on chromosome 3PS in the genetic background of wheat. *A. cristatum* chromatin is visualized by red color, whereas wheat chromosomes are counterstained with DAPI (**c** and **d**). Scale bar is 10 μm

**
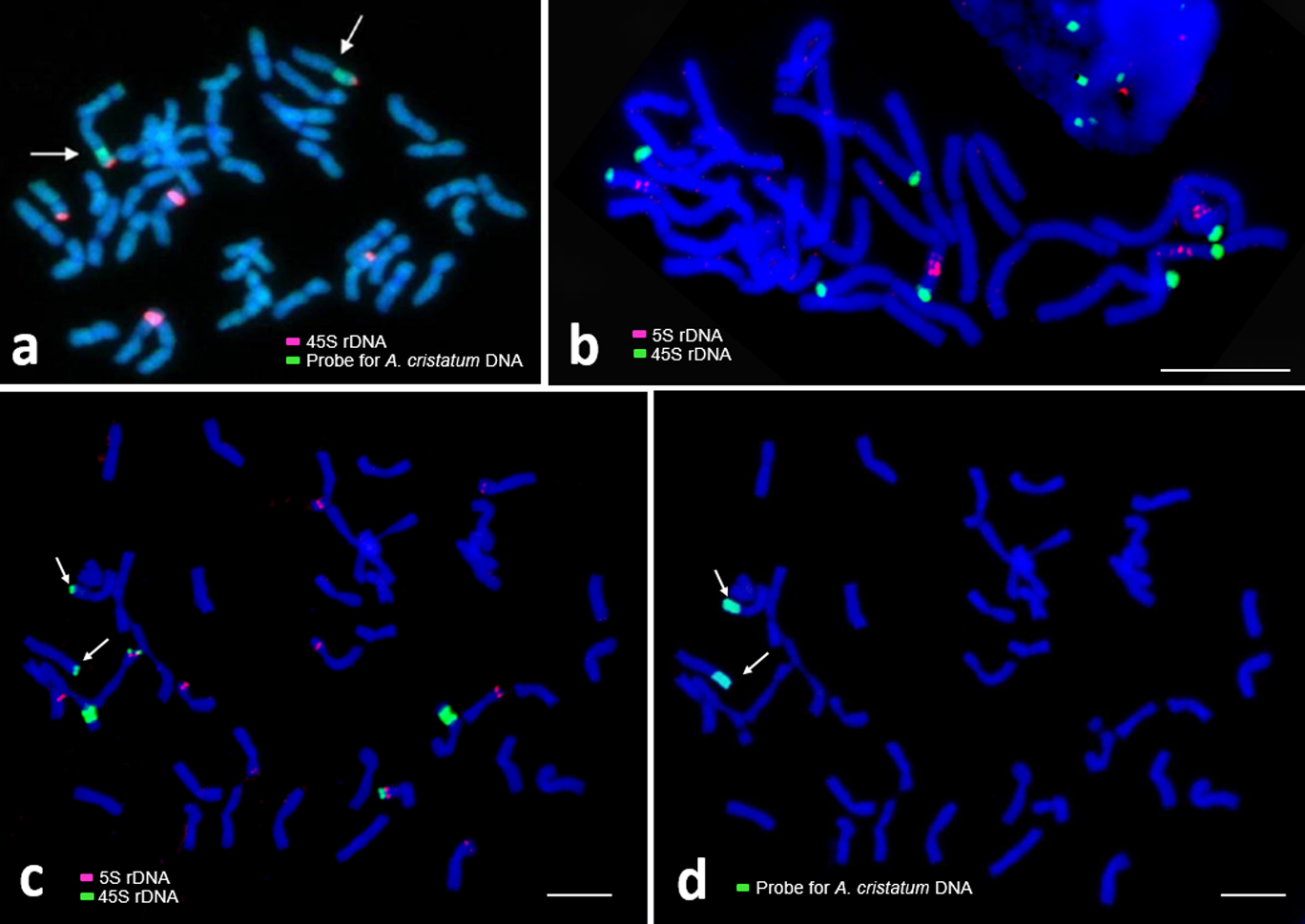
**

**Supplementary Fig. S3** FISH and GISH for identification of 1PS arm in the background of wheat. **a**) unknown *A. cristatum* chromosome short arm (arrows) translocated to wheat chromosome arm 1BL, and detected by GISH using DNA from *A. cristatum* (green) and 45S rDNA signals (red) by Ochoa et al. (2015). **b**) Probe for 45S rDNA (green) localized on the short arms of four pairs of chromosomes of the auto-tetraploid *A. cristatum* PI22297, which was used for the development of the translocation, two pairs of these chromosomes were characterized by sub-terminal singles of 5S rDNA (red), the chromosomes were identified by Said et al. (2018) as 1P and 5P, respectively. **c**) The 45S rDNA (green) was detected on the translocated *A. cristatum* chromosome arm (arrows) in the background of wheat, while 5S rDNA (red) was absent. **d**) GISH using DNA from *A. cristatum* (green) distinguished the translocated arm (arrows). The chromosomes were counterstained with DAPI (blue). Scale bar is 10 μm
