# Supplementary material for: Uncovering homeologous relationships between tetraploid Agropyron cristatum and bread wheat genomes using COS markers
Source: Theor Appl Genet. 2019 Jul 16;132(10):2881–98. doi: 10.1007/s00122-019-03394-1 (PMC6763527; doi:10.1007/s00122-019-03394-1)
Supplement: Supplementary file 2 — Supplementary material 2 (DOC 93 kb) [file 122_2019_3394_MOESM2_ESM.doc]

**Uncovering homoeologous relationships between tetraploid *Agropyron cristatum* and bread wheat genomes using COS markers**

**Mahmoud Said^1,2^, Alejandro Copete Parada^3^, Eszter Gaál^4^, István Molnár^1,4^ Adoración Cabrera^3^, Jaroslav Doležel^1^, Jan Vrána^1*^**

^1^Institute of Experimental Botany, Center of the Region Haná for Biotechnological and Agricultural Research, Šlechtitelů 31, CZ-78371 Olomouc, Czech Republic

^2^Field Crops Research Institute, Agricultural Research Centre, 9 Gamma Street, Giza, 12619 Cairo, Egypt

^3^Genetics Department, ETSIAM, Agrifood Campus of International Excellence (ceiA3), University of Córdoba, 14071, Spain

^4^Agricultural Institute, Centre for Agricultural Research, Hungarian Academy of Sciences, Martonvásár, Hungary

*Jan Vrána

Email: [vrana@ueb.cas.cz](mailto:vrana@ueb.cas.cz)

Tel: +420 585 238 720

**Supplementary Table S1.** Frequency (%) of plants with various chromosome composition in progenies of wheat CS-*A. cristatum* lines (based on summarized data from chromosome counting and FISH).

| **Genotype** | ***N*** | **Frequency (%) of plants with various chromosome numbers** | | | | | | |
| --- | --- | --- | --- | --- | --- | --- | --- | --- |
|  |  | 44 | 43 | 42 + 1 + 1 telo | 42 + 2 telos | 42 + 1 telo | 42 + 1 iso | 42 |
| **1P** | 26 | 92 | 8 | - | - | - | - | - |
| **2P** | 28 | 86 | 14 | - |  |  |  |  |
| **3P** | 23 | - | - | 9 | - | 17 | - | 74 |
| **4P** | 26 | 92 | 8 | - | - | - | - | - |
| **5P** | 30 | 93 | 7 | - | - | - | - | - |
| **6P** | 23 | 96 | 4 | - | - | - | - | - |
| **2PS** | 21 | - | - | - | 47 | 10 | - | 43 |
| **2PL** | 23 | - | - | - | 87 | 13 | - | - |
| **3PS** | 22 | - | - | - | 77 | 23 | - | - |
| **4PS** | 23 | - | - | - | 83 | 17 |  |  |
| **5PL** | 23 | - | - | - | 91 | 9 | - | - |
| **6PS** | 20 | - | - | - | 90 | 5 | 5 | - |
| **6PL** | 22 | - | - | - | 100 | - | - | - |
| **CST-1PS·1BL** | 21 | - | - | - | 100 | - | - | - |
| **Total** | 331 |  |  |  |  |  |  |  |

*N* = number of plants analyzed, for which frequencies were calculated

**Supplementary Table S2.** Mean values for spike agronomic traits comparing Chinese Spring (CS) and wheat-*A. cristatum* addition and translocation lines

| **Genotype** | **Spike length (cm)** | **Spikelets/spike** | **Seeds/spike** | **Fertility( Seeds/spikelets)** |
| --- | --- | --- | --- | --- |
| **Common wheat CS** | 7.9 ± 0.7^cde^ | 20.0 ± 1.2^fg^ | 68.6 ± 4.0^a^ | 3.44 ± 0.11^a^ |
| **CS-1P** | 4.8 ± 0.3^g^ | 11.9 ± 0.6^h^ | 13.0 ± 1.6^g^ | 1.10 ± 0.10^h^ |
| **CS-2P** | 9.0 ± 0.4^b^ | 22.1 ± 0.8^def^ | 8.2 ± 1.8^g^ | 0.37 ± 0.03^i^ |
| **CS-3P** | 8.1 ± 0.2^cd^ | 21.7 ± 0.7^ef^ | 51.0 ± 3.1^cd^ | 2.36 ± 0.07^cd^ |
| **CS-4P** | 7.5 ± 0.4^def^ | 18.3 ± 1.2^g^ | 14.2 ± 1.9^g^ | 0.78 ± 0.06^h^ |
| **CS-5P** | 8.0 ± 0.6^cd^ | 21.1 ± 1.2^ef^ | 57.4 ± 3.1^bc^ | 2.72 ± 0.08^bc^ |
| **CS-6P** | 7.1 ± 0.2^ef^ | 24.8 ± 0.8^bc^ | 69.2 ± 2.7^a^ | 2.79 ± 0.06^b^ |
| **CS-2PS** | 8.1 ± 0.2^cd^ | 22.1 ± 0.8^def^ | 46.6 ± 3.9^d^ | 2.11 ± 0.06^de^ |
| **CS-2PL** | 8.5 ± 0.4^bc^ | 28.7 ± 0.9^a^ | 57.6 ± 4.5^bc^ | 2.01 ± 0.06^def^ |
| **CS-3PS** | 7.9 ± 0.4^cde^ | 21.9 ± 0.9^def^ | 35.4 ± 3.2^e^ | 1.62 ± 0.09^fg^ |
| **CS-4PS** | 8.0 ± 0.4^cd^ | 24.9 ± 1.5^bc^ | 68.4 ± 3.9^a^ | 2.76 ± 0.13^b^ |
| **CS-5PL** | 7.0 ± 0.0^f^ | 21.8 ± 1.1^ef^ | 37.8 ± 3.8^e^ | 1.74 ± 0.06^efg^ |
| **CS-6PS** | 8.0 ± 0.5^cd^ | 24.0 ± 0.7^cd^ | 36.4 ± 4.4^e^ | 1.52 ± 0.06^g^ |
| **CS-6PL** | 7.0 ± 0.4^f^ | 22.7 ± 0.4^cde^ | 63.8 ± 3.3^ab^ | 2.81 ± 0.07^b^ |
| **CST-1PS·1BL** | 11.3 ± 0.5^a^ | 26.3 ± 1.1^b^ | 22.8 ± 3.1^f^ | 0.87 ± 0.07^h^ |

Values within a column followed by the same letter are not significantly different (*P* ≤ 0.05)

**Supplementary Table S3.** Molecular markers indicating intrachromosomal rearrangements in *Agropyron cristatum* relative to hexaploid wheat

| Marker | Homoeologous chromosome group | Chromosome arm location | |
| --- | --- | --- | --- |
|  |  | Wheat | *A. cristatum* |
| c740349 | 1 | S | L |
| c743346 | 1 | S | L |
| c767527 | 3 | L | S |
| TR63 | 3 | L | S |
| c740051 | 4 | AL, BS, DL | S |
| TR647 | 4 | AL, BS, DS | S |
| c763059 | 4 | AS, BS, DL | S |
| c756721 | 5 | L | S |
| BE445667 | 6 | S | L |
| c724446 | 6 | L | S |
